# Exosomes in Ageing and Motor Neurone Disease: Biogenesis, Uptake Mechanisms, Modifications in Disease and Uses in the Development of Biomarkers and Therapeutics

**DOI:** 10.3390/cells10112930

**Published:** 2021-10-28

**Authors:** Ekene Anakor, Laura Le Gall, Julie Dumonceaux, William John Duddy, Stephanie Duguez

**Affiliations:** 1 Northern Ireland Center for Stratified/Personalised Medicine, Biomedical Sciences Research Institute, Ulster University, Derry-Londonderry BT47 6SB, UK; Anakor-E@ulster.ac.uk (E.A.); l.gall@ucl.ac.uk (L.L.G.); j.dumonceaux@ucl.ac.uk (J.D.); w.duddy@ulster.ac.uk (W.J.D.); 2 NIHR Biomedical Research Centre, Great Ormond Street Institute of Child Health, Great Ormond Street Hospital NHS Trust, University College London, London WC1N 1EH, UK

**Keywords:** extracellular vesicle, exosome, CNS, neuromuscular disease, neurodegenerative disease, ageing, biomarkers, therapeutic

## Abstract

Intercellular communication between neurons and their surrounding cells occurs through the secretion of soluble molecules or release of vesicles such as exosomes into the extracellular space, participating in brain homeostasis. Under neuro-degenerative conditions associated with ageing, such as amyotrophic lateral sclerosis (ALS), Alzheimer’s or Parkinson’s disease, exosomes are suspected to propagate toxic proteins. The topic of this review is the role of exosomes in ageing conditions and more specifically in ALS. Our current understanding of exosomes and exosome-related mechanisms is first summarized in a general sense, including their biogenesis and secretion, heterogeneity, cellular interaction and intracellular fate. Their role in the Central Nervous System (CNS) and ageing of the neuromotor system is then considered in the context of exosome-induced signaling. The review then focuses on exosomes in age-associated neurodegenerative disease. The role of exosomes in ALS is highlighted, and their use as potential biomarkers to diagnose and prognose ALS is presented. The therapeutic implications of exosomes for ALS are considered, whether as delivery vehicles, neurotoxic targets or as corrective drugs in and of themselves. A diverse set of mechanisms underpin the functional roles, both confirmed and potential, of exosomes, generally in ageing and specifically in motor neurone disease. Aspects of their contents, biogenesis, uptake and modifications offer many plausible routes towards the development of novel biomarkers and therapeutics.

## 1. Introduction

Mechanisms involving chemical messengers, the extracellular matrix, gap junctions, tunnelling nanotubes and extracellular vesicles exist in cells for communication and exchange of bioactive materials including organelles, genetic materials, pathogens and misfolded proteins [1]. Based on characteristics of their production and release from cells, classes of extracellular vesicles include exosomes, microvesicles and apoptotic bodies [2]. The use of the term “exosomes” can be traced to a 1981 paper where the writers proposed that exfoliated membrane vesicles be referred to as exosomes [3]. In 1983, two independent studies reported that the maturation of reticulocytes into erythrocytes involved the release of transferrin receptors via 50 nm vesicles [4,5]. Four years later, the term exosome was used by Rose Johnstone to refer to vesicles released into the extracellular space following fusion of the multivesicular bodies (MVBs) with the plasma membrane [6]. For the purpose of this review, and in line with the International Society for Extracellular Vesicles (ISEV) designation [7], the term small EVs (sEVs) will be used interchangeably with exosomes. Exosomes can be released by different cell types in vitro and can be detected in biological fluids in both pathological and physiological contexts. 

Intercellular communication between neurons [8] and their surrounding cells [9,10,11] in the central nervous system occurs through the secretion of soluble molecules or release of vesicles such as exosomes containing neuroprotective factors in the extracellular space [12,13], participating in the maintenance of the brain homeostasis [14,15]. Under neurodegenerative conditions associated with ageing, such as amyotrophic lateral sclerosis (ALS), Alzheimer’s or Parkinson’s disease, exosomes are suspected to propagate toxic proteins [16,17,18,19]. 

After summarizing exosome biogenesis, exosome heterogeneity and their fate and impact on recipient cells, this review will then focus on the role of sEVs in ageing conditions and more specifically on Amyotrophic Lateral sclerosis (ALS), a multisystemic condition [20] that is associated with ageing. Thereafter, we will discuss whether exosomes could be used as therapeutic tools and/or as biomarkers for ALS.

## 2. Exosome Biogenesis and Secretion

**Figure 1:**
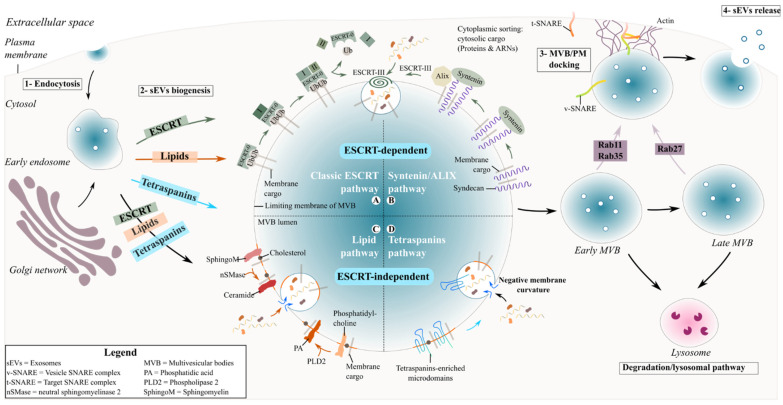
Biogenesis and secretion of exosomes. Schematic representation of exosome formation and release in the extracellular space. (**1**) Exosomes are produced as ILVs by inward budding of the endosomal membrane and accumulate in the lumen of the endosome. (**2**) Several mechanisms are involved in the biogenesis of exosomes such as ESCRT protein-, lipid-raft- and tetraspanin microdomain-dependent pathways. Whether one or multiple pathways are required simultaneously by one population of MVBs, or if each pathway is specific to one population of MVB, is still not clear. ESCRT-dependent pathways: (**A**) the most described mechanism involved in the biogenesis of exosomes is the ESCRT-dependent pathway requiring proteins of the ESCRT family. Specific transmembrane ubiquitinated cargo is recruited and clustered at the MVB membrane by the ESCRT-0 complex, subsequently binding to the ESCRT-I structure. The ESCRT-II complex is activated and together with ESCRT-I will create and/or stabilize the vesicle neck. Finally, ESCRT-III and its associated proteins will drive neck constriction. (**B**) The second ESCRT-dependent biogenesis pathway is the syntenin/ALIX pathway. The formation of syndecan-enriched microdomains leads to syndecan cleavage and the formation of syntenin/syndecan complexes that interact with ALIX. The syntenin–syndecan–ALIX complex then favours the recruitment of the ESCRT-III complex to support the MVB membrane curvature and abscission. ESCRT-independent pathways: (**C**) Ceramide- and phosphatidic acid-dependent pathways are based on the formation of lipid-rafts where sphingomyelin is converted to ceramide or phosphatidylcholine is converted to phosphatidic acid. The ceramide- and phosphatidic acid-enriched rafts induce the inward curvature of the MVB membrane. (**D**) Similarly, tetraspanin-enriched microdomains can induce a negative curvature in the MVB membrane. (**3**) MVBs will either fuse with lysosomes for degradation or with the plasma membrane, which will consequently release exosomes into the extracellular space (**4**). Several proteins have been identified in the transport and fusion of the MVB to the plasma membrane, such as proteins from the Rab protein family and SNARE complexes.

During exosome biogenesis, early endosomes mature into late endosomes where intralumenal vesicles (ILVs) are formed and accumulate in their lumen. The process of exosome formation includes (1) the clustering of sorted cargo at the membrane of the MVBs, forming microdomains, and (2) subsequent membrane curvature and fission of vesicles. Generally, the fate of MVBs is to fuse with lysosomes for degradation of their content. However, MVBs can also be targeted to the plasma membrane of the cell where ILVs are released into the extracellular space as exosomes upon membrane fusion [21]. The role of endosomal sorting complexes required for transport (ESCRT) proteins in exosome biogenesis has been investigated using a variety of approaches such as proteomics and RNA silencing screening analysis [22,23]. The depletion of the four ESCRT complexes involved in exosome biogenesis did not totally abrogate exosome formation, indicating the existence of other mechanisms [23,24]. Two different pathways are described for sEV formation (Figure 1), summarised below.

### 2.1. ESCRT-Dependent Mechanism

The ESCRT proteins can cluster into four complexes: ESCRT-0, I, II and III [25] and are involved in the sorting of ubiquitinated cargo into ILVs (Figure 1). The ESCRT-0 complex is composed of HRS (Hepatocyte growth factor-regulated tyrosine kinase substrate) and STAM (Signal transducing adapter molecule) proteins and is recruited to the endosomal membrane via ubiquitinated cargo and phosphatidylinositol 3-phosphate (PI3P). HRS recognizes ubiquitinated protein–ubiquitin acting in this context as a targeting signal for the specific incorporation of molecules in ILVs- and binds to PI3P [26,27]. The HRS/STAM complex recruits ESCRT-I via TSG101/VPS28 (two components of the ESCRT-I complex) to the endosomal membrane and forms an ESCRT-0/ESCRT-I complex. The ESCRT-I complex contains one copy each of TSG101, VPS28, VS37 [26] and MVB12. Its recruitment at the endosomal membrane is enhanced by ubiquitinated transmembrane cargo. Similar to ESCRT-0, it is also involved in the clustering of selected ubiquitinated cargo into microdomains and mobilizes the ESCRT-II complex. The ESCRT-II complex is a heterodimer comprising one copy each of VPS36 and VPS22 and two VPS25 subunits [28]. Together with the ESCRT-I complex, the ESCRT-II complex initiates the negative curvature of the emerging ILV at the MVB membrane and the uptake of cytosolic cargo [27]. Finally, the association of ESCRT-I and –II recruits the ESCRT-III complex at the ILV biogenesis site via ALIX or through a direct interaction with VPS25 from ESCRT-II. The components of the ESCRT-III complex polymerize into filaments after recruitment at the MVB membrane with two protein complexes, VPS2-VPS24 and VPS20-SNF7 [28,29]. ESCRT-III inside the nascent neck of the ILV leads to the closure and detachment of vesicles containing specific cargo within the MVB lumen [26,27].

Another ESCRT-mediated exosome biogenesis involves the interaction of ESCRT-III/ALIX with the transmembrane proteoglycan receptor, syndecan, and its binding partner syntenin [30]. Syndecans assemble at the MVB membrane, followed by cleavage of the syndecan auto-repulsive domain. They remain clustered at the membrane allowing syntenin to bind to the syndecan bundle. Consequently, syntenin interacts with ALIX, recruiting the ECRT-III unit with VPS4 and leading to endosomal membrane inward budding and abscission. ALIX initiates a de-ubiquitination step that occurs before the incorporation of proteins into the ILV and before the closure of the latter [26].

### 2.2. ESCRT-Independent Mechanisms

#### 2.2.1. Lipid-Mediated Biogenesis

Exosomes are enriched in cholesterol, sphingolipids and ceramide. ESCRT-independent ceramide-mediated exosome biogenesis requires the conversion of sphingomyelin into ceramide through neutral sphingomyelinase 2 and the conversion of phosphatidylcholine into phosphatidic acid (PA) by Phospholipase D2 (Figure 1) [31]. Subsequently, ceramide and PA generated at the limiting membrane of the MVBs form a cone-shaped structure that may contribute to the negative curvature of the endosomal membrane, leading to inward budding and ultimately the formation of ILVs that are released as sEVs [32]. 

#### 2.2.2. Tetraspanin-Mediated Biogenesis

The tetraspanin family are regulators of non-ESCRT dependent exosome biogenesis (Figure 1). Tetraspanin proteins possess four transmembrane domains resulting in two extracellular and three intracellular regions [33]. Tetraspanin proteins are glycosylated to various degrees [33], forming oligomers and a protein-enriched microdomain at the plasma membrane [34]. The role of glycosylation modifications of tetraspanins is still unknown but it possibly contributes to tetraspanin complex formation [35]. Due to their cone-shaped conformation and their ability to cluster into microdomains, tetraspanins could induce inward budding of the late endosomal membrane and exosome formation [36]. Tetraspanins are highly enriched within exosomes and have been specifically used as exosomal biomarkers over the years (see Table 1). Among them CD63, CD81, CD82 or CD9 are particularly used as exosome markers.

### 2.3. Release of Exosomes in the Extracellular Space

MVBs are directed either to lysosomes for degradation or to the plasma membrane to release exosomes into the extracellular space (Figure 1). The secretion requires cytoskeleton network-associated molecular motors, small GTPases and fusion machinery including SNARES and tethering proteins [2,80]. Small GTPase Rab and SNARE protein families are particularly required for the transport of MVBs towards the cell periphery and their docking and fusion with the plasma membrane [81]. Exosome secretion is mediated by different Rabs that are preferentially associated with early (Rab11 [82] and Rab35 [83]) or late (Rab27) endosomes. The subsequent fusion of the MVB-limiting membrane with the plasma membrane requires soluble factors (NSF and SNAP), SNAP-attachment protein receptor (SNARE) protein complexes and protein from the synaptotagmin family [81,84]. The v-SNARE complex in the vesicle membrane interacts with the t-SNARE complex located at the cell membrane. The SNARE proteins form bridges between opposing membranes that brings them sufficiently close to induce fusion of both lipid layers. Consequently, ILVs present in MVBs are released into the extracellular space as exosomes.

## 3. Heterogeneity in the Exosome Population

Exosomes are heterogeneous and have been categorized according to size [84], morphology [85,86] or buoyant density [87,88,89]. Heterogeneity can be attributed to exosome biogenesis occurring at different locations along the endosome, plasma membrane or the apical or cortical regions of the cell [85,87,88,89,90,91,92,93].

### 3.1. Exosome Membrane Composition

Lipid bilayer membrane delimited-exosomes are enriched in ceramide, cholesterol, phosphatidylserine, phosphatidic acids, sphingomyelin, fatty acids, prostaglandins, leukotrienes and sphingolipids that provide rigidity and structural stability [94]. The exosome membrane also contains tetraspanin membrane organisers-CD9, CD81, CD63 and CD82 [27,81,84,95,96], transmembrane proteins (lipid-rafts or the tetraspanins), fusion proteins (cytoskeletal, annexins or flotillin) and adhesion molecules (integrins and lactadherins) [97,98,99]. The membrane composition varies depending on the biogenesis pathway, leading to the release of different exosome subpopulations [89,99]. Table 1 summarizes the proteins commonly detected by Western blot or immunostaining in exosomes secreted by different cell types in the central nervous system and by the muscle cells.

### 3.2. Exosome Lumen Content

The lumen composition of exosomes varies depending on the site of formation and the biogenesis pathway [89,99] and on the physiological and pathological context. In this section, the general content and sorting processes will be described. The composition of exosomes in the context of ageing and ALS will be described in Section 5 and Section 6.

#### 3.2.1. Proteins

The most abundant proteins identified in the lumen of exosomes include proteins required for the biogenesis and function of exosomes such as ESCRT and associated proteins (ALIX and TSG101, see Table 1) [27,84,100] that are shared across different exosome subpopulations. The exosome lumen also contains functional enzymes such as lipolytic enzymes implicated in intraluminal vesicle formation and eicosanoid biosynthesis [101,102]. Protein cargo sorting inside sEVs is controlled by specific machinery with post-translational modifications (PTM) acting as a sorting signal [99]. 

Protein ubiquitination is an important sorting signal involved in ESCRT protein machinery recruitment. Likewise, ESCRT-independent mechanisms involve PTMs such as SUMOylation, phosphorylation, citrullination or oxidation [99,103]. For example, α-Synuclein, a presynaptic neuronal protein linked genetically and neuropathologically to Parkinson’s disease, once SUMOylated, is incorporated into sEVs, released into the extracellular milieu and readily internalised by other cells in the central nervous system, transferring toxic alpha synuclein oligomers in a cell-to-cell manner [104].

While ubiquitination and SUMOylation are involved in targeting proteins to MVBs and their subsequent release via exosomes, others such as acetylation and ISGylation drive modified proteins into MVBs directed towards lysosomal degradation [105], suggesting that PTM could serve as a mechanism to direct potentially toxic proteins into sEVs for clearance.

It is noteworthy that not all exosomal protein cargo is modified and that not all modified proteins are sorted into exosomes. Other processes such as ESCRT-independent pathways involving the tetraspanins (eg CD63), ceramide and lipid raft domains also have a role in protein cargo sorting in sEVs [106].

To date, it is not clear whether all protein sorting machineries overlap or are completely independent and/or specific to a sub-type of ILVs. Further studies are required to better understand the sorting of specific PTM proteins into the exosomes.

#### 3.2.2. Nucleic Acid

Depending on the cell type and the state of the secreting cell, the lumen of exosomes contains nucleic acids including genomic and mitochondrial DNA [107] and different types of RNAs [108]. The exact mechanism(s) by which nucleic acids, especially miRNAs, are loaded into exosomes are not fully understood, but potential modes of sorting have been postulated [109]. To illustrate exosome heterogeneity in RNA content, a single copy of a given miRNA (e.g. miR-126, miR-223 and miR-720) was observed in only one exosome out of one hundred [90], an indication that the RNA sorting process and content in sEVs might not be reproducible.

Numerous studies report the presence of diverse nucleic acids in sEVs such as microRNAs (miRNA), messenger RNAs (mRNAs), transfer RNA (tRNA), vault RNAs, circular RNAs, long non-coding RNAs (lncRNAs) and small nucleolar RNA (snoRNA) [110,111], as well as mitochondrial and genomic DNA [9,112]. The RNA composition of sEVs is currently being explored in depth. Similar to protein cargo sorting, the mechanisms of RNA sorting have been widely studied, especially for miRNAs (see [113,114] for review). Numerous mechanisms for miRNA sEV sorting have been identified, such as (1) the miRNA-induced silencing complex (miRISC) pathway that co-localises with MVBs and involves proteins such as AGO2 [109]; (2) the ESCRT-independent pathway requiring neutral sphingomyelinase 2 [115]; (3) miRNA motifs and sumoylated heterogeneous nuclear ribonucleoproteins (hnRNPs)-dependent sorting that requires the presence of a GGAG (EXOmotifs) or GGCU sequence (hEXO motifs) to bind and load miRNAs into sEVs [103,116]; (4) membrane proteins involved in ESCRT biogenesis such as Vps4A, which modulates the sorting of microRNAs into sEVs [117,118]. Other mechanisms of sorting of miRNAs and other types of RNA species such as mRNAs, lncRNAs, tRNAs and circRNAs that include SUMOylation [103], and raft-based microdomains requiring the presence of a lipid-bilayer binding motif within specific RNA sequences [114,119]. In addition, other RNA-binding proteins including YB-1, NSUN2, MEX3C, Major Vault Protein 4(MVP4), La protein, MTR4, and Anexin-2 can sort RNA species into sEVs by recognizing and binding specific RNA sequences [106]. 

The mechanisms sorting DNA species into sEVs have not been directly explored and remain relatively unknown, although the ESCRT family proteins and mitochondria-derived vesicles generated in response to oxidative stress and targeted to the endolysosomal system may play a role in sorting [120,121]. Interestingly, it has been suggested that most of the DNA associated with sEVs is not localised within the intraluminal space but on the outer membrane of the vesicles [122,123].

### 3.3. Heterogenous Buoyant Properties

Buoyant properties will vary according to the exosome composition and size, with small vesicles (~60 nm) reaching their density equilibrium faster than large vesicles (~100 nm) [87,88]. Two types of sEVs were identified following sucrose gradient ultracentrifugation: low-density exosomes with density of 1.12–1.19 g/mL and size distribution between 75–200 nm and high-density exosomes with density of 1.26–1.29 g/mL and size distribution < 100 nm [88]. Table 2 collates data from different studies of exosomes secreted by neuronal and glial cells, which suggest that most sEVs from these cells present a diameter between 70–75 nm, and a low density (between 1.13–1.16 g·mL^−1^). sEVs secreted by adult muscle cells have a higher diameter, around 100 nm.

Although heterogeneous exosome populations are secreted by cells, most of the studies analyse exosomes as bulk isolates, masking vesicle subpopulations and the physiological or pathological effects of these subpopulations.

## 4. Exosomal Interaction with Recipient Cells and the Fate of Exosomes

**Figure 2:**
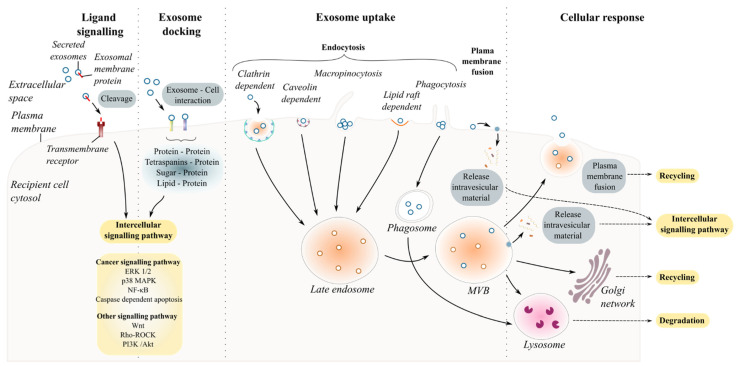
Exosome and recipient cell communication. Schematic diagram summarizing exosome–cell interactions. Once secreted into the extracellular space, exosomes mediate cellular responses via distinct pathways. Exosomes are described as messengers carrying functional cargo that can alter the physiology of the targeted cell once internalized. However, exosome uptake and/or direct contact with the targeted cell to mediate cell–exosome communication are not always required. Indirect interaction between secreted vesicles and cells is possible through soluble ligand signalling. Exosomes carry transmembrane proteins on their surface, accessible for cleavage by proteases to produce soluble forms of proteins that interact with specific receptors on the plasma membrane of recipient cells. Upon reaching the intended recipient cell, exosomes can dock at the plasma membrane. Following the anchorage of the vesicles, the activation of multiple distinct intercellular signalling pathways might occur via ligand/receptor interaction, also known as juxtacrine signalling. Most studies, however, describe the internalization of exosomes by the recipient cells. Exosome uptake involves several mechanisms of endocytosis including: (1) clathrin-dependent mechanism, (2) caveolin-mediated endocytosis, (3) macropinocytosis, (4) phagocytosis, and (5) lipid-raft dependent endocytosis. To release their content into the cytoplasm, secreted exosomes can directly fuse with the plasma membrane. The vesicle–cell interaction generates distinct cellular responses. Communication mediated by ligand signalling, Juxtacrine signalling or direct fusion with the plasma membrane possibly results in intercellular signalling pathway activation. On the other hand, once endocytosed by the cells, exosomal contents are systematically released to the endocytic compartments and are more likely to undergo degradation via fusion of endosomes with lysosomes. Some vesicles, however, have been described to escape degradation by back fusion of the exosomes containing MVB with the PM or by transport of exosomes towards the Golgi apparatus. MVB: Multivesicular bodies.

Understanding whether exosomes communicate with the recipient cell in a specific and controlled or stochastic process is the first step in unravelling exosome-cell interaction [125,126]. Intercellular communication mediated by secreted exosomes occurs either by direct interaction with recipient cell with or without the uptake of exosomes and/or indirect interaction facilitated by cleaved transmembrane ligands (proteins or lipids). This section addresses the types of interactions between exosomes and the recipient cell (Figure 2).

### 4.1. Ligand–Receptor Interaction (Cell-Surface and Exosome-Surface Receptors)

Exosome–recipient cell interaction requires a combination of specific molecules present on the surfaces of the cell and on the exosome including proteins (glycoproteins, integrins or tetraspanins), sugar (heparan sulfate proteoglycans) and lipids. Table 3 reports some of the ligands on the surface of exosomes and the targeted cells that are known to interact with each other.

Several studies have investigated how exosomes could specifically target different cells under physiological and pathological conditions [136,143,144]. Among the list of known ligand–receptor interactions, protein–protein interactions are the most abundant for sEVs. For example, the pre-treatment of ovarian cell-derived exosomes with proteinase K or trypsin to degrade exosomal transmembrane protein abolished their uptake by cancer cells [136,145,146]. Inhibiting specific interactions using antibodies or soluble ligands prior to treatment of cells with exosomes has enabled the discovery of many specific ligand–receptors involved in exosome uptake [147]. The various specific ligand–receptors suggest that some sEVs may target specific cells and/or may have different effects on different cells. Once an exosome docks at the surface of the recipient cells, four scenarios can occur. The exosome binds to cell surface receptors eliciting intracellular responses in the recipient cell [148]; it fuses with the plasma membrane releasing its contents directly into the cytosol [149]; it is internalized by the recipient cell via the endosome machinery [150,151]; or it crosses the cell and is re-released intact to target other cell types [152]. 

### 4.2. Indirect Communication: Soluble Ligand Mediated Signalling 

Exosomes can mediate intercellular communication without direct contact with the recipient cell by producing soluble ligand resulting from the cleavage of transmembrane protein–ligand that will then interact with its receptor at the surface of the targeted cell and activate multiple signalling pathways (Figure 2–ligand signalling). For example, as part of the complement activation pathway, CD46 has been identified as one of the mediators of complement resistance of malignant cells by inactivating C3b and C4b molecules [148]. CD46 can shed from the tumour cells via exosomes and is cleaved by metalloproteinases to produce soluble forms in ovarian cancer cells [148]. 

### 4.3. Fusion of Exosomes with the Plasma Membrane

The fusion of the exosome and recipient cell membranes requires several events including insertion of hydrophobic fusogenic proteins into the recipient cell, lipid reorganisation, protein restructuring to fusion-competent forms and membrane dimpling [149]. Syncytin-1 and -2, exosomal transmembrane proteins, bind to syncytin-specific receptors, MFSD2a and ASCT2 located on the recipient cell surface [153]. Interestingly, these surface proteins are known to be expressed by glial and neuronal cells, with syncytin-1 expressed in microglial cells [154] and associated with neuroinflammation [155], MFSD2a expressed by the endothelial cells at the blood–brain barrier [156] and ASCT2 expressed by both astrocytes and neurons [157,158]. Following binding, the exosome membrane fuses with and is inserted within the plasma membrane, resulting in the release of exosomal contents into the cytoplasm of targeted cells [149].

The pH of the extracellular space also plays an important role in the vesicle–cell fusion process, as exosome fusion was enhanced by acidic pH while pre-treatment with proton pump inhibitors, which reduces extracellular space acidity, reversed this phenomenon [159]. The brain extracellular pH is locally and tightly regulated by astrocytes following significant release of acid by neurons [160]. An imbalanced local pH regulation may not only affect neuronal functions, but also the interactions between neurons and sEVs.

### 4.4. Endocytosis

Exosome interaction with recipient cells can occur through the endocytic pathways including clathrin- or caveolin- dependent endocytosis, macropinocytosis, phagocytosis, and lipid-raft mediated endocytosis.

#### 4.4.1. Clathrin-Mediated and Caveolin-Dependent Endocytosis 

Various studies have highlighted the possibility of exosomes to be internalized by energy-dependent mechanisms involving the cytoskeleton of the recipient cells [138,161]. A well-known pathway is clathrin-mediated endocytosis [162]. The deformation of the cell membrane induced by the clathrin protein leads to the formation of inward buds growing into a larger vesicle that will mature and pinch off (see [163] for review). The exosomal content can then be delivered into the recipient cells, as observed with rat pheochromocytoma tumour cell-derived exosomes absorbed by bone marrow-derived mesenchymal stromal cells (BMSCs) via clathrin-dependant endocytosis and delivering miR21 [150]. 

Caveolae are well known to be involved in the endocytic pathway and could be involved in the absorption of circulating sEVs. They are invaginations in the plasma membrane enriched in glycoproteins and cholesterol [164]. Three caveolins, cavelonin-1,-2 and -3, can form oligomeric complexes that are stabilized by cavin proteins [165]. Caveolin rafts are then internalized by the cell through dynamin activity [147]. However, while epithelial cells uptake exosomes via clathrin and caveola-dependant pathways [151], HCT116 cells [166] and BMSCs [150] are not able to internalize exosomes via caveolae. These different studies highlight that the recipient cells and the exosome origin may influence the pathway used for the absorption of circulating vesicles.

#### 4.4.2. Macropinocytosis and Phagocytosis 

Exosomes can be secreted as a cluster [67], thus affecting incorporation through the classic clathrin- and caveolin-dependent mechanisms. In this context, both pinocytosis and phagocytosis pathways can form large vacuoles [167] and can engulf large exosome clusters and aggregates. Despite their similarity, phagocytosis and pinocytosis occur through two distinct cellular machineries. 

Macropinocytosis is characterized by the formation of ruffled extensions from the plasma membrane around the extracellular space including the extracellular fluid and components that will be further internalized by the cell. Macropinocytosis machinery requires multiple mediators such as PAK1 kinase, rac1, ras and src, cholesterol, cytoskeleton actin protein and a Na+/H+ exchanger [167]. It occurs constitutively and requires the protein-dependent formation of cytoskeletal actin [52].

Phagocytosis relies on the association between the receptor from the plasma membrane and the vesicle’s ligand [52]. The co-localization of exosomes with phagolysosome maturation markers (e.g., LBPA, Rab7 and LAMP proteins) strongly suggests the ability of macrophages to internalize exosomes via phagocytosis, forming large exosome-containing vacuoles that are targeted towards lysosomes [168]. This requires the formation of membrane invagination around the targeted cargo to be internalized and involves the actin cytoskeleton, PI3K, dynamin and phosphatidylserine (PS) [147,169]. Phagocytic cells mainly use phagocytosis for the capture of exosomes [168].

#### 4.4.3. Lipid-Raft-Mediated Endocytosis 

The colocalization of exosomes with a lipid raft marker pointed towards the role of lipids in the uptake of exosomes and was confirmed when exosome uptake was successfully inhibited with cholesterol-depletor, Methyl-β-cyclodextrin added to glioblastoma cells, or when lipid-raft dependent endocytosis inhibitor drugs were used [147,170]. Lipid rafts are formed by cholesterol and sphingolipid-rich microdomains and are rich in protein receptors [147]. However, lipid-raft mediated endocytosis may represent a small portion of exosome uptake as only a small region of the plasma membrane is rich in sterols and sphingolipids, and this region may also be involved in various cellular processes [171]. 

### 4.5. Fate of Exosomes within Targeted Cells

The fate of sEVs following entry into the recipient cell is still being investigated and not fully understood. Three destinies can be observed: (1) recycling/degradation, (2) delivery of functional content and (3) crossing the cells and being released intact to other cell types where they can exert their action.

Following internalisation, exosomes are most likely integrated into the endocytic pathway and those directed to the late endosome are degraded within the lysosome with the release of materials that can be used by the recipient cell [172]. However, internalised sEVs maybe be able to escape degradation via recycling of endosomes or the trans-Golgi network. More specifically, the late endosome can accumulate vesicles that contain molecules not destinated to be degraded by lysosomes [173], and will release functional content into the cytoplasm. Consequently, the functional nucleic acids and proteins delivered to the recipient cells could have an impact on cellular pathways causing cellular reprogramming, epigenetic changes or modulation of the phenotype [110].

The recipient cell may not be the final destination for sEVs. As observed in neurons, sEVs can hijack the endosomal pathway and be transported with endogenous exosomes to neighbouring or distant cells. Using a microfluidics setup, PKH-67-labelled exosomes from the brain of Tau transgenic rTg4510 mice were internalised by the endosomes of cultured mcherry-cd9 labelled neurons that re-released a mixed exosome population (red and green labelled exosomes) to neighbouring neurons [152]. This highlights the property of exosomes to engage in long-distance communication with intact sEV content containing toxic proteins, as observed in sEVs implicated in neurodegenerative diseases [152].

## 5. Exosome-Induced Signaling in CNS; Role in Ageing and in the Neuromotor System

sEVs can play important roles in neuronal plasticity, neuron-glia communication, muscle-neuron communication, homeostasis, protection from cellular stress and synaptic regulation. Presynaptic and post-synaptic secretion of neuronal exosomes mediates neuron–neuron and neuron–glia communication [8,67,174]. Exosome-mediated neuron–neuron communication is involved in neuronal growth and differentiation [37] and suppression of dendritic growth [175] as well as homeostatic regulation of synaptic plasticity [176,177,178]. Neuronal exosomes can also promote microglial synaptic pruning of neurites by upregulation of pro-phagocytic genes [179], while exosomes secreted by glial cells can protect and ensure neuronal integrity and survival [174,179,180,181]. For example, sEVs secreted by oligodendrocytes not only regulate myelination and neuronal survival [13,51], but also act as a metabolic support under stress conditions [12] via the delivery of enzymes (catalase, SOD1) and phosphorylation of signaling proteins such as CREB, GSK-3α/β, GSK-3β and JNK within neurons [13]. Astrocyte-derived exosomes promote neuron survival and protection under oxidative stress conditions, containing molecules such as apolipoprotein E [182], apolipoprotein D [183], neuroglobin [65] and gap junction protein [184] that are associated with neuronal repair, survival and anti-apoptosis. Similarly, microglia-derived exosomes are important for neuronal homeostasis and provision of metabolic support [11,39]. N9-cell lines and primary microglia cultures secrete exosomes containing enzymes associated with glycolysis and lipid metabolism that may supplement neuronal metabolic support [11]. 

Skeletal muscle-derived exosomes are secreted by both myoblasts and myotubes [69,185] and may be involved in neuronal cell survival [186], myogenesis and muscle regeneration [73,187] as well as in myoblast differentiation [71,79] or during muscle ageing [188]. C_2_C_12_ myotube-derived exosomes reduced myoblast proliferation and induced differentiation while negatively regulating Sirt1 expression in C_2_C_12_ myoblasts, further supporting the existence of myoblast-myotube crosstalk mediated by exosomes [71,79]. 

These studies highlight the contributions of exosomes derived from motor neurons, glial cells and skeletal muscles to neuromuscular system functioning and the cross talk that is persistent within and between different cell types. 

### 5.1. Impact of Ageing on Cell–Cell Communication

Neuronal and non-neuronal cells including the skeletal muscles are impacted by normal ageing. For example, ageing motor neurons that are post-mitotic undergo an analogous senescence requiring P53 activation that results in cellular stress, aberrant neuronal health and an enhanced vulnerability to further pathological insult [189]. Skeletal muscle undergoes structural and functional changes with ageing, with resident skeletal muscle adult stem cells (satellite cells) exhibiting age-associated loss of regenerative capacity due to defects in activation, proliferation and self-renewal [188,190]. Alteration of the intrinsic properties of ageing cells may affect their local niche [191], affecting the cell secretome and thus communication from cell to cell [188] and may have a role in ageing-related processes such as neuroinflammation [155], inflammaging [192] or neurodegeneration [193], processes also known to be involved in ALS. 

sEV biogenesis and secretion are altered with ageing, leading to an increased secretion of sEVs with smaller size and modified miR profiles that may have an impact on macrophage phagocytosis [194]. sEVs also present a dramatic increase in the expression of exosomal markers CD63 and LAMP2 with ageing [195]. 

### 5.2. Secretion of sEVs by Senescent Cells

The degree of increase in sEV secretion is dependent on the cell type origin and their senescence level [196]. In senescent cells, sEV biogenesis and secretion are upregulated by p53 acting as a transcription factor (see [197] for review) and by the Ras-related RAB family of small GTPase genes [198,199]. Furthermore, P53 upregulates neutral sphingomyelinase-2 [200], while DNA damage, which is a key trigger for the induction of senescence, activates ceramide biosynthesis that results in biogenesis of senescent-associated sEVs [201]. 

The accumulation of senescent cells with age can influence the release and contents of circulating sEVs. Senescence-associated secretory phenotype (SASP) components such as interleukins, intercellular adhesion molecule 1 and Cell-free telomeric repeats containing RNA (cfTERRA) are present in sEVs from different senescent cell types [202], and miRNAs involved in senescence pathways have been identified in sEVs, with a capacity to affect cellular functions in the body [194] in an autocrine and/or paracrine fashion. 

Although extracellular vesicles are involved in senescence and ageing, evidence for the role(s) of sEVs in physiological ageing and neurodegeneration is in its infancy, and the mechanisms and/or signaling involved in specific tissues such as neural and skeletal tissues still need to be elucidated. For example, primary human myoblasts undergoing premature senescence showed a five-fold increase in sEV secretion, with gene expression analysis showing a four-fold increase in transforming growth factor-β (TGF-β) within secreted sEVs [203]. These aged skeletal muscle-derived sEVs increased the expression of senescence markers and reduced proliferation in endothelial cells [203]. Furthermore, sEVs from aged C_2_C_12_ myotubes show age-associated significant enrichment in miR-34a that induces cellular senescence in bone marrow mesenchymal stem cells [204] and in miR-29b-3p that is efficiently transferred to neuronal cells, inhibiting genes associated with neuronal differentiation while decreasing neurite length and outgrowth [205]. Recently it has been suggested that sEVs containing SASP components activate transcription factors involved in the canonical NF-κB pathway and are reliant on the IKK Complex, a central regulator of NF-κB activation to drive senescence [206].

Overall, as senescence drives ageing and DNA damage accumulation is widespread in aged brains and is higher in pathological brains, the presence of SASP components within isolated sEVs suggests a role of EVs in communication with the cellular microenvironment and possible contribution to age-related tissue and organ dysfunction.

Interestingly, while the concentration of sEVs in peripheral circulation is increased in age-related diseases [207], there is no clear evidence as to whether senescence-associated sEVs in peripheral circulation increase with age. While no correlation was found between blood sEV concentration and healthy human aging as well as frailty status [208], the sEV concentration in plasma decreasing with advancing age could be due to increased internalization by leukocytes [209]. Together, these studies suggest that while senescent cells in vitro may provoke an increase in sEV secretion, circulating sEVs either remain the same or are decreased with ageing. 

### 5.3. Proteins and miRNAs Associated with Senescence Contained within sEVs 

Ageing affects the RNA and protein composition of sEVs. Galectin-3 is reduced in plasma sEVs of elderly subjects [210], and several sEV-associated miRNAs have been implicated in brain ageing. When sEVs from young rats that are enriched in miR-129 are applied to aged rats, there was increased myelination and a reduction in the functional decline of the brain [211]. 

Senescent cell-derived sEVs that are enriched in miR-23a-5p and miR-137 can bring about telomere dysfunction, confer anti-apoptotic properties and induce cellular senescence in recipient cells [212,213].

### 5.4. sEV Therapeutics in Ageing

sEVs mediate the systemic delivery of biologics that counteract age-associated functional decline in target tissues including the hypothalamus and hippocampus [214]. Extracellular nicotinamide phosphoribosyl transferase (eNAMPT) is a nicotinamide adenine dinucleotide (NAD+) biosynthetic enzyme that declines with age in humans. The administration of sEVs isolated from the plasma of young mice and containing eNAMPT improved the wheel-running activity and increased lifespan of aged mice, suggesting the utility of young sEVs as a potential anti-ageing intervention [215]. Similarly, hypothalamic neural stem cell (NSC)-derived sEVs possess anti-ageing effects that are mediated in part by miRNAs. The administration of NSC-derived sEVs to the hypothalamic third ventricle of ageing animal models reduced hypothalamic inflammation and slowed down the age-associated detrimental outcomes [211].

sEVS extracted from human iPSCs [216], embryonic stem cells [217], primary fibroblasts of young human donors [218], mesenchymal stromal cells [219] and human embryonic stem cell-derived MSCs [220] have all been described to attenuate senescence and cell aging in vitro and in vivo and to extend health span. Together, these studies suggest that sEVs could be beneficial for age-related pathologies and can be used as a potential therapeutic strategy. 

## 6. Exosomes in Neurodegenerative Disease Associated with Ageing: ALS

Motor neuron disorders are a heterogeneous group of diseases characterised by the progressive and fatal degeneration of upper and/or lower motor neuron [221,222]. Amyotrophic lateral sclerosis (ALS) is the most frequent of the motor neuron diseases, with reported incidence varying between 1 and 2.6 per 100,000 persons per year in different populations [222], with approximatively 10% of cases being familial and 90% being sporadic cases [20,223]. 

The aetiology of ALS is not fully understood with cellular, environmental and genetic factors thought to play a role [20]. The most frequent gene mutations associated with ALS are copper- and zinc-containing antioxidant superoxide dismutase 1 (*SOD1*), Fused in Sarcoma (*FUS*), *C9orf72* and TAR DNA-binding protein 43 (*TARDBP/TDP43*) [222]. Several pathways associated with cellular dysfunction are often described in the nervous or muscle tissues of ALS patients including glutamate toxicity, oxidative stress, mitochondrial dysfunction, axonal transport impairment, protein aggregation, endoplasmic reticulum stress, abnormal RNA processing and neuroinflammation [224]. The contribution of exosomes to ALS pathology by propagating misfolded proteins or toxic aggregates is increasingly being investigated [225,226,227], as well as their use as prognostic or diagnostic biomarkers.

### 6.1. Detection of ALS Proteins in Exosomes: Potential Role of Exosomes in the Propagation of ALS

Aggregation of misfolded proteins may participate in disease propagation [228]. While the exact mechanism(s) for the spread of neurodegeneration is not fully understood in ALS, extracellular secretion of misfolded or aggregated proteins via exosomes may contribute to ALS pathogenesis. In this context, the role of exosomes as carriers of toxic elements to neighbouring and distant cells is increasingly being investigated.

Mutant proteins associated with ALS including SOD1, Valosin-containing protein (VCP), FUS, TDP43, other RNA-binding proteins and dipeptide repeats (DPRs) resulting from *C9orf72* expansions are present in exosomes derived from cells overexpressing these proteins [227,229,230]. While overexpression studies provide a model to study the consequences of disease-associated proteins and the possible relationship between misfolded or mutant protein secretion and contribution to pathology, it is unclear whether protein overexpression causes the preferential accumulation of ALS-associated proteins within exosomes in these studies.

SOD1 is responsible for the clearance of reactive oxygen species (ROS) in cells. Exosomes containing mutated or misfolded SOD1 are reportedly secreted by motor neurons, astrocytes and microglia [43,229,230,231] and are detected in the brain and spinal cord of human SOD1^G93A^ mice [18]. Mutated or misfolded SOD1 decorates the surface of exosomes [18,232] and can transfer these toxic elements to healthy cells, as observed with exosomes carrying HuSOD1^G127X^ or misfolded SOD1 [232], suggesting the capacity for mutant and/or misfolded SOD1 containing exosomes to participate in the spread of ALS.

VCP, an AAA-ATPase involved in ubiquitin-dependent protein degradation and autophagy and also associated with ALS [233], has been detected in exosomes secreted by astrocytes overexpressing SOD1^G93A^ [230]. 

The DNA/RNA-binding proteins including TDP43, FUS and Matrin 3 can be observed in exosomes and are known to be involved in various aspects of RNA metabolism and processing, with mutations in these proteins affecting pathways in RNA processing [234]. TDP43 cytoplasmic inclusions are a pathological hallmark of ALS [235] with exosomes containing oligomeric TDP43 or its C-terminal fragments causing cytoplasmic TDP43 redistribution and aggregation in recipient cells [236,237] as well as neuronal soma-to-soma and bi-directional (anterograde and retrograde) axonal TDP43 transmission [226]. Similar to SOD1, the presence of TDP43 was observed on the membrane of secreted vesicles. Taken together, these studies corroborate the possible involvement of exosomal TDP43 or its fragments in intercellular trafficking and spread of toxicity, while raising questions about the significance of SOD1 and TDP43 on the surface of exosomes. 

The presence of the FUS protein within exosomes suggests a contribution to FUS pathology and that exosomes may mediate propagation of mutated FUS and hence, ALS toxicity. Kamelgarn’s group revealed an interaction between FUS, Matrin-3 and hnRNPA1 (FUS interaction partners) and the presence of wild and mutant FUS within exosomes from neuronal cells [238]. FUS and its partner RPL5 and caprin-1 were also detected in exosomes secreted by ALS skeletal muscle stem cells that were toxic toward human iPSC-derived motor neurons [70].

Finally, the dipeptide repeat species (DPRs) generated by the hexanucleotide repeat expansions in *c9orf72* [239] can be detected in exosomes secreted by DPR-transfected neuronal cells and were present as cytoplasmic aggregates when transferred to healthy cortical neurons [227]. 

In light of these data, exosomes could be seen as a protective proteostasis mechanism that ensures cell survival by conveying toxic materials including misfolded proteins out of the cell [240]. However, several studies highlight protein cargo in exosomes as a mechanism for cell–cell spread of toxicity and potential propagation in ALS and may explain ALS pathogenesis [225,226,227,230,241]. 

### 6.2. Potential Role of Exosomes to Modify Pathways in Recipient Cells in ALS

MiRNAs are small non-coding RNAs responsible for the precise control of transcriptional and post-transcriptional gene regulation, highlighting their epigenetic potential [242] with roles in neuronal communication [243], myogenesis and muscle homeostasis [242]. Two ALS genes, *FUS* and *TARDBP*, are essential for miRNA biogenesis and pre-miRNA processing [234], with mutations in these genes correlating with dysregulated RNA processing and metabolism in ALS cells or tissues [224]. RNA dysregulation has been implicated in the disease with numerous studies supporting a role for miRNAs in ALS [243,244,245,246,247,248].

MiRNAs packed within exosomes exhibit increased stability and protection from RNAse [249] and can be transferred between cells [110], suggesting that exosomal transfer of miRNAs could represent an epigenetic mechanism causing changes within ALS pathways and contributing to disease pathology. Exosome miRNA expression profiles are functionally different from those of the parent cells [109], with significant differences observed in the miRNA profiles of mouse astrocyte-derived exosomes compared to astrocytes [62].

The capacity for exosomal miRNAs to modify ALS pathways in recipient cells was demonstrated in cells expressing SOD1 and *c9orf72* mutations [68,250]. For example, miR-124 is enriched in exosomes derived from motor neurons expressing mutant SOD1. These exosomes promote the expression of pro-inflammatory miRNAs (miR-155) while reducing the expression of anti-inflammatory miRNAs, consequently leading to microglia proinflammatory M1 activation [68,251,252]. On the other hand, Varcianna and colleagues recently identified 13 dysregulated miRNAs including miR-494-3p that are associated with axonal guidance and maintenance pathways in sEVs derived from *C9orf72* astrocytes [250]. Furthermore, miR-494-3p was the most dysregulated miRNA and is associated with the regulation of semaphorin 3A-an axon guidance protein that is increased in the motor cortex and decreased in the spinal cord of ALS patients [253,254].

Thus, ALS exosomes are a possible conduit for dysregulated miRNAs that could contribute to epigenetic or functional changes in near or distant recipient cells, facilitating the neurodegenerative process seen in ALS including inflammation and motor neuron death.

## 7. Use of Exosomes in Therapeutic Strategies for Neurodegenerative and Neuromuscular Conditions

### 7.1. Unmodified Exosomes as Therapeutics in Motor Neuron Disease

In their native state and without modification of either surface receptors or proteins, exosomes from different cell types possess reparative, regenerative and restorative effects in different diseases [255]. Exosomes from healthy adipocytes [256,257,258] rescued the ALS phenotype observed in SOD1-mutated neuronal cells with the following: 1- restitution of the mitochondrial respiratory function [257] and mitochondrial transcription factor (p-CREB and PGC-1α) expression [256]; 2- prevention of oxidative damage in SOD1^G93A^ [256,257,258] SOD1^G37R^ and SOD1^A4V^ [258] neuronal cells; and 3- a significant decrease in SOD1 aggregates two and six days after exosome treatment [256]. Proteomics analysis of healthy adipose-derived exosomes revealed the presence of 189 proteins implicated in Bcl-2α protein upregulation, cell adhesion and negative regulation of the apoptotic process, suggesting they could be neuroprotective when applied to mutated SOD1 neurons [259]. Similar results were obtained with exosomes derived from healthy mesenchymal stem cells (MSCs), neural crest-derived human dental pulp stem cells (hDPSC) and human bone-marrow mesenchymal stem cells (hBM-MSC) acting through anti-apoptotic and anti-necrotic mechanisms as well as by enhancing the endogenous neuronal survival factors of recipient cells [260]. Importantly, the number of cell passages prior to exosome isolation appears to be important for exosome cargo and function [261] with an inverse relationship established between passage number and exosome neuroprotection [262]. Exosomes derived from early passages (P3 and P5) of rat bone MSCs were more efficient at neuroprotection compared to later passages (P8), this being mediated via anti-apoptotic, anti-necrotic and antioxidant mechanisms [262].

Recently, and as a first proof of in vivo use of exosomes in ALS, adipose-derived stem cell (ASC-) exosomes administered intravenously and intranasally at the clinical onset of the disease to hSOD1^G93A^ mice improved motor performance, protected spinal MN and muscle fibres from degeneration, preserved the neuromuscular junction by slowing axonal detachment from muscles and reduced astroglial activation [263]. Interestingly, intranasal administration demonstrated the capacity for the exosomes to target injured areas of the ALS mice brain, indicating possible tissue tropism [264]. Surprisingly, at time points > 17 weeks (late phase of the disease), the neuroprotection and improved motor performance associated with ASC-exosomes disappeared, irrespective of administration route, raising questions relating to exosome dosage and exosome effectiveness at a late stage of the disease. 

### 7.2. The Possibility to Use Modified Exosomes as Therapeutic Vehicles: Lessons from Other Neurodegenerative and Neuromuscular Conditions

Exosomes are attractive as vehicle systems for small therapeutic molecules and/or biomolecules including nucleic acids and proteins because of their lipid nature, presence of specific surface ligands (CD11b and CD18 receptors, integrins, tetraspanins) and ability to cross the blood–brain barrier [265]. When compared to other drug delivery systems, exosomes have the distinct advantages of blood–brain barrier penetrance, longer duration in systemic circulation, tissue specificity that minimizes unwanted toxicity or off-target effects, stability of content, desirable biocompatibility and minimal toxicity issues [266]. Techniques such as fusion expression, exosome membrane surface display and anchoring platforms have been used to attach peptides and biological ligands of interest to adhesion molecules, tetraspanins or integrins on exosome surface to ensure targeted delivery and enhanced uptake into desired cells [265,267,268]. 

In diseases characterized by motor neuron degeneration, modified exosomes have been used to deliver specialized molecules to specific cell types. A popular example and the earliest use of modified exosome therapeutics is the rabies viral glycoprotein (RVG)-exosomes isolated from genetically engineered cells expressing lamp2b fused with a neuron-specific peptide and used to deliver functional cargo to organs expressing acetylcholine receptors [126]. The administration of RVG-exosomes containing β-site amyloid precursor protein cleaving enzyme (BACE1) siRNA in wild-type mice significantly reduced mRNA and protein levels of BACE1, a key target for therapeutic inhibition of β-amyloid production in Alzheimer’s disease [269]. In addition, RVG-exosomes containing α-synuclein specific DNA aptamers [270] or anti-α-synuclein short hairpin RNA mini circles [271] decreased gene and protein expression of misfolded α-synuclein for prolonged periods in animal models of Parkinson disease and alpha-synucleinopathy, respectively. These modified exosomes were preferentially targeted to specific cells (neurons and glial cells) and regions of the brain, releasing their content and mediating a decrease in aggregated proteins, suggesting the utility of this strategy to target neurodegeneration. Furthermore, skeletal muscle targeting was successfully achieved in a murine model of Duchenne Muscular Dystrophy using modified exosomes bearing CP05 (a specific CD63 exosomal anchor peptide) and M12 (muscle targeting peptide) and containing dystrophin splice–correcting morpholino oligomer with a reported increase in dystrophin expression in skeletal muscles and functional rescue without associated oligomer toxicity [272]. 

## 8. Exosomes as Molecular Biomarkers for ALS

Presently, no single diagnostic test exists for the diagnosis of ALS, with clinicians relying on a combination of history, physical examination, neuroimaging, electrodiagnostic and laboratory findings [20,222]. ALS shares certain overlap with other neurodegenerative diseases, which makes diagnosis difficult with a reported lag of 12 months between the onset of symptoms and neurological diagnosis [222]. Biomarkers provide opportunities to improve diagnosis, monitor disease progression, gauge prognosis, aid patient stratification and response to therapy [20,273], and exosomes may be well suited for these roles. Molecular biomarker development for ALS is at an all-time high with investigated biomarkers cutting across proteins, miRNAs, mRNAs and metabolites from cerebrospinal fluid (CSF) and blood (extensively reviewed in [20]). 

The investigation of sEVs as diagnostic and/or prognostic biomarkers for ALS has been increasingly investigated during the last decade. Disease pathology affects the composition of exosomes [274] as well as their secretion and/or accumulation [275,276]. Advantageously, proteins and RNAs associated with a disease and enclosed in exosomes exhibit stability in biological fluids as they are protected from degradation by the double membrane structure of the sEVs and can be stored for long periods before analysis [277,278]. This would suggest that the content could be sensitively detected if appropriate isolation protocols that ensure near-pure sEVs are utilised. 

Exosomes and their contents as molecular biomarkers for ALS have been investigated in cerebrospinal fluid [279,280], plasma ([281,282,283] and serum [284] (Table 4). One of the earliest studies observed, in serum-derived exosomes from patients, that miR-27a-3p was significantly downregulated when compared to controls [284]. In addition, proteomics analysis of exosomes from cerebrospinal fluid identified 334 proteins including Novel INHAT Repressor (NIR) that were increased in sEVs from sporadic ALS cohorts [279]. NIR protein is associated with nucleolar stress, a major contributor to *c9orf72*-linked neurodegeneration [285]. An apparent drawback with using sEVs from biological fluids is that they may suffer from “contamination” arising from plasma proteins, which is contingent on the sEV isolation protocol used.

A different approach to plasma or serum biomarker studies is to consider plasma and/or serum as a mix of exosomes secreted from multiple cell sources and implementing a strategy that allows for the isolation of exosomes from a specific cell population such as neurons, glial cells or even myoblasts. Using LCAM1 immunoprecipitation after exosome isolation from plasma to obtain neuron-specific exosomes, microarray analysis revealed 30 dysregulated exosomal miRNAs even though a small sample size was used [283], while a follow-up study using a larger sample size and validated by qPCR identified eight miRNAs that consistently and significantly differentiated ALS cohorts from healthy controls [286]. Similarly, biotinylated glutamine aspartate transporter (ACSA-1) antibody immunoprecipitation following exosome isolation from plasma yielded astrocyte-derived sEVs with interleukin 6 (IL6) content elevated in sALS cohorts and positively correlated with rate of disease progression and disease duration less than 12 months [282]. Although not entirely specific to ALS, as interleukin 6 is elevated in other neurodegenerative diseases, it could still be a useful marker for neuroinflammation and disease progression. Table 4 summarizes the studies discussed above and highlights the source of biological fluids, exosome parameters, methods for analysis of the respective biomarkers and significance of the studies.

**Table 4:** Studies evaluating sEVs as molecular biomarkers for diagnosis in Amyotrophic lateral sclerosis (ALS).

Biofluid	sEV Isolation Technique	Exosome Parameters (Size, Exosome Marker).	Study Design	Exosome Origin & Analysis	Study Summary	Possible Biomarker	Ref
Plasma	Polymer-based precipitation and immunoaffinity purification using anti-CD-171	102 nm, CD63(+), TSG101(+), Calnexin (−) Neural Markers: L1CAM (+), NCAM 1 (+), MAPT (+), GRIA 1 (+), PLP 1 (+)	HC: 20 ALS: 20 Type: Not disclosed Age ≥ 18 yrs. old ALSFRS-R > 25 and FVC score ≥ 60%.	Neuron-derived exosomes Next generation sequencing (NGS) analysis, then downstream qPCR	3 miRNAs downregulated and 5 miRNAs upregulated consistent and significant in ALS cohorts	miRNA fingerprinting for early ALS diagnosis	[286]
Plasma	Polymer-based precipitation and immunoaffinity purification using anti-CD-171.	150 nm CD81(+), CD63(+) SNAP25 (+) Synaptophysin (+) [Neuron specific markers].	HC: 5 ALS: 5 Type: Sporadic ALS Age and Sex matched.	Neuron-derived Exosomes Microarray analysis.	30 differentially regulated miRNAs in ALS. miRNA upregulated (ALS): 13 miRNA downregulated (ALS): 17	miRNAs within neuron-derived exosomes might be clinically advantageous in ALS diagnosis.	[283]
Plasma	Polymer-based precipitation and immunoprecipitation with biotinylated mouse anti-human glutamine aspartate transporter (ACSA-1) antibody.	100 nm CD63 (+), Calnexin (−)	Recruitment: HC: 40 ALS: 39 For Study: HC: 12 ALS: 15 Type: Sporadic ALS (Bulbar onset:12; Limber onset: 28). ALSFRS-R: 39.83 ± 1.08 Age and Sex matched.	Astrocyte-derived Exosomes Enzyme-linked immunosorbent assay (ELISA)	Interleukin-6 (IL-6) levels increased in all ALS subgroups with no significant difference. Positive correlation between IL-6 levels and disease progression rate but not with total ALSFRS-R scores, diagnosis delay or patient age. For ALS < 12 months, Positive correlation between IL-6 levels in ADEs from ALS patients and rate of disease progression	IL-6 possible biomarker? Need for further studies and larger sample size.	[282]
Plasma	Heat Shock Protein- Vn96 synthetic peptide isolation followed by Centrifugation.	Nil parameters presented	HC: 12 ALS: 14 Type: Sporadic ALS (5M, 7F) Familial ALS (1M, 1F) ALSFRS-R: 26.23 ± 8.09	droplet digital PCR-based miRNA quantification	27 differentially regulated miRNAs in ALS. miR-15a-5p and miR-15a-5p/miR-181b-1-5p combination show diagnostic potential. miR-193a-5p distinguishes PALS with low and high ALSFRS-R scores.	miR-15a-5p and miR-193a-5p can be aid diagnosis and monitor ALS progression.	[281]
Serum	Polymer-based precipitation OR membrane affinity isolation	CD63 (+)	HC: 20 ALS: 10 Nil information on age matched or ALFRS score	Serum Exosomes Quantitative real-time PCR (qRT-PCR)	Downregulated expression of miR-27a-3p in ALS group that was statistically significant.	miR-27a3p as a reference for ALS diagnosis.	[284]
CSF	Sample concentration followed by Size Exclusion Chromatography and/or Ultracentrifugation.	30–150 nm, CD81 (+), CD9 (+)	HC (iNPH): 3 ALS: 3 Type: Sporadic ALS Age and Sex matched. ALSFRS-R: 42.00 ± 1.00	CSF-exosomes (exosome-enriched fractions from CSF) Proteomics	334 proteins were identified including NIR (Novel INHAT Repressor) which was significantly increased in exosomes.	NIR as ALS biomarker and role in pathogenesis	[279]
CSF	Centrifugation	186 nm ± 70.4 nm CD9 (+), CD81 (+) Flotillin-1 (+)	HC: 4 ALS: 4 Type: Sporadic ALS Age and Sex matched. ALSFRS-R: 41–45 Disease duration: 0.5–5 years	Neuronal-derived Exosomes Next-generation sequencing and qRT-PCR	543 genes were significantly changed between HC and ALS groups. Genes upregulated (ALS):133 Genes downregulated (ALS): 410	CUEDC2 (most increased exosomal mRNA in CSF from ALS group)	[280]

This table highlights the biological fluid used; sEV isolation protocol and parameters as well as the method of analysis used in identifying the proteins or nucleic acids contained within the sEVs. The significance of the study is also highlighted. +: marker expressed in sEV studied, −: marker absent in the sEV studied.

## 9. Conclusions

Taken together, these studies highlight the diverse set of mechanisms underpinning the functional roles, both confirmed and potential, of exosomes, generally in ageing and specifically in motor neurone disease. Aspects of their contents, biogenesis, uptake and modifications offer many plausible routes towards the development of novel biomarkers and therapeutics. Native exosomes are increasingly implicated in pathological mechanisms, and both modified and unmodified exosomes have potential in the treatment of diseases associated with neurodegeneration or skeletal muscle dysfunction, making them potentially suited for deployment in motor neurone disease.

Despite their reported and documented roles in the pathology of ageing-related diseases, emerging studies highlight the neuroprotective and regenerative properties of sEVs in improving ageing and functional or cognitive decline. Although the use of exosome-based therapy in clinics is limited, preclinical studies would suggest that sEVs from young cohorts hold beneficial effects in age-associated diseases that could be translated. It would be interesting to investigate the capacity for modified exosomes to target toxic or misfolded proteins (SOD1, TDP43, FUS, dipeptide repeats) implicated in ALS by serving as conduits for new pharmacological agents or biologicals to improve therapeutic outcomes. As our understanding of the complex pathology of ALS and contribution of exosomes increases, the use of modified exosomes presents an exciting opportunity for new therapeutics in ALS. Similarly, as the role of exosomes in this disease is increasingly explored, the potential for therapeutic targeting of neurotoxic exosomes should be tested. 

In addition, exosomes are a promising and potential source of biomarkers for ALS prognosis and patient stratification. Since exosomes secreted by motor neurons, glial and inflammatory cells, and skeletal muscles enter systemic circulation (plasma or CSF), exosome-associated proteins or nucleic acids that reflect the status of these cells will be particularly useful considering the multicellular and multisystem nature of ALS. It will be interesting to see more studies focused on isolating cell-specific exosomes from biological fluids of ALS cohorts. However, technical issues relating to the isolation process that retains sEV purity and integrity, contaminant elimination, cohort study size, validation and cost-effectiveness need to be addressed.

## Figures and Tables

**Table 1:** Exosomes released by cells in the central nervous system and by the neuromuscular system.

Exosome Origin	ALIX	CD9	CD63	CD71	CD81	CD82	Flotillin-1	Hsp70	Hsp90	Lamp1	Lamp2	Rab7	Rab11	Tsg101	Calnexin	Ref
Cortical neurons	+++		+				+++							++		[8,37,38,39]
Microglial cell	++	++++++++	+++++++		+		+++	+		+++	+	+	+	+		[11,40,41,42,43,44,45,46,47,48,49,50]
Oligodendrocytes	++++++	+						++						+++	− − − −	[10,12,13,51,52,53]
Schwann cells	+++	++	++++				+	++++	+					+++		[54,55,56,57,58,59]
Astrocytes	+++		++++		+		+++	++	+					++++		[9,60,61,62,63,64,65,66]
Hippocampal neurons	+													+		[67]
Motor neurons	+		+				+									[68]
Skeletal muscle cells	+++++++++	+	+++++	+	+++++	++	+	++						++++++	− − −	[69,70,71,72,73,74,75,76,77,78,79]

Non-exhaustive list of different cell types secreting exosomes including neuronal cells (cortical neurons and neuroglial cells) and skeletal muscle cells. The classic exosome markers used to identify and characterize isolated vesicles (ALIX, CD9, CD63, CD81, Flotillin, Hsp70 and Tsg101) are shown. Each “+” or “−” sign represents a paper reporting sEVs harbouring typical exosomes markers (“+”) or absence of the endoplasmic reticulum marker, calnexin (“−”).

**Table 2:** Vesicle heterogeneity in size and variability in buoyant densities in isolated sEV populations.

Exosome Origin	Ref	Exosome Marker	Density (g.mL^−1^)													
			1.07	1.08	1.09	1.1	1.11	1.12	1.13	1.14	1.15	1.16	1.17	1.18	1.19	1.2
Rat primary cortical neurons	[8]	ALIX							100							
Murine oligodendrocytes	[10]							55								
Oligodendrocyte cell line	[12]					95										
Oligodendrocyte cell line	[52]										75					
Murine neuroglial cells	[124]	Tsg101							70							
Oligodendrocyte cell line	[52]										75					
Murine neuroglial cells	[124]	Hsc70/ Hsp70							70							
Oligodendrocyte cell line	[12]									95						
Murine neuroglial cells	[10]	Flotillin							70							
Oligodendrocyte cell line	[52]															
Human embryonic myotubes	[72]	CD63					70									
Human embryonic myotubes	[72]	CD81	70				70									
Human embryonic myotubes	[72]	CD9					70									70
Human adult myotubes	[69]	CD63					100									
Human adult myotubes	[69]	CD82								100						

This table summarizes studies conducted on vesicles originating from cell culture. Exosomes are ranked depending on their buoyant properties (in sucrose in g/mL) and associated with the mean vesicle size in nm as measured by electron microscopy (EM) or nanoparticle tracking analysis (NTA) methods. Exosome markers used to identify exosome populations on density gradient separation for each study are listed in the second column. The grey backgrounds indicate the flotation of vesicles.

**Table 3:** Summary of ligand–receptor interaction in exosomes/cell communication.

Exosome Ligand		Target Cell Ligand	Ref
Glycoproteins	Fibronectin	Heparin sulfate proteoglycans (HSPGs)	[127]
	Fibronectin	Integrins	[128]
	ICAM (CD54)	LFA-1	[129,130,131]
	MUC1	DC-SIGN	[132]
Integrins	β1 and β2 integrins	ICAM-1	[133]
	β1 and β2 integrins	Collagen-I	
	β1 and β2 integrins	Fibronectin	
	Integrin α4β1	Fibronectin	[134]
	αvβ3 / αvβ5 integrins	MFG-E8	[135]
	CD47	SIRPα	[136]
Lectin	C-type lectin	Mannose-rich C-type lectin receptor	[137]
	Galectin 5	Glycoproteins (CD7, α5β1-integrin, or laminin)	[138]
	Galectin 9	Tim 3	[139]
Tetraspanins	Tspan8-CD49d	ICAM-1 (CD54)	[140]
Lipid rafts	Phosphatidylserine	Tim-1/4	[96]
	Phosphatidylserine	MFG-E8	[135]
	Phosphatidylethanolamine	MFG-E8	
	Annexin 2	Lipid raft domain	[141]
Sugar	α2,3-linked sialic acids	sialoadhesin (CD169)	[142]

Known ligand/receptor interactions are listed and categorized according to the molecular origin of the ligand. ICAM: InterCellular Adhesion Molecule, LFA-1: Lymphocyte Function-associated Antigen 1, MFG-E8: Milk Fat Globule-EGF factor 8 or lactadherin protein, SIRPα: SIgnal Regulatory Protein α, DC-SIGN: Dendritic Cell-Specific Intercellular adhesion molecule-3-Grabbing Non-integrin C type lectin receptor.

## Abbreviations

ADEs: Astrocyte derived exosomes; ALSFRS-R: ALS Functional Rating Scale-revised; anti-CD171: anti-human CD171 also called L1CAM; CD9: CD9 Molecule; CD63: CD63 Molecule; CD81: CD81 Molecule; CSF: Cerebrospinal fluid. CUEDC2: CUE Domain Containing 2; FVC: Forced vital capacity; GRIA 1: Glutamate Ionotropic Receptor AMPA Type Subunit 1; HC: Healthy Control; iNPH: idiopathic normal-pressure hydrocephalus; L1CAM: L1 Cell Adhesion Molecule; MAPT: Microtubule Associated Protein Tau; NCAM1: Neural Cell Adhesion Molecule 1; PALS: persons diagnosed and living with ALS; PCR: Polymerase chain reaction; PLP 1: Proteolipid Protein 1; SNAP25: Synaptosome Associated Protein 25; TSG101: Tumour Susceptibility 101; vn96: vn96 peptide.
